# Histone deacetylase inhibitors for cancer therapy: An evolutionarily ancient resistance response may explain their limited success

**DOI:** 10.1002/bies.201600070

**Published:** 2016-09-22

**Authors:** John A. Halsall, Bryan M. Turner

**Affiliations:** ^1^ Chromatin and Gene Expression GroupInstitute of Cancer and Genomic SciencesUniversity of BirminghamBirminghamUK

**Keywords:** cancer, chromatin, deacetylase, epigenetic drugs, evolution, histone modification

## Abstract

Histone deacetylase inhibitors (HDACi) are in clinical trials against a variety of cancers. Despite early successes, results against the more common solid tumors have been mixed. How is it that so many cancers, and most normal cells, tolerate the disruption caused by HDACi‐induced protein hyperacetylation? And why are a few cancers so sensitive? Here we discuss recent results showing that human cells mount a coordinated transcriptional response to HDACi that mitigates their toxic effects. We present a hypothetical signaling system that could trigger and mediate this response. To account for the existence of such a response, we note that HDACi of various chemical types are made by a variety of organisms to kill or suppress competitors. We suggest that the resistance response in human cells is a necessary evolutionary consequence of exposure to environmental HDACi. We speculate that cancers sensitive to HDACi are those in which the resistance response has been compromised by mutation. Identifying such mutations will allow targeting of HDACi therapy to potentially susceptible cancers.

Also see the video abstract here.

**Abbreviations**
CTCL: Cutaneous T‐Cell Lymphoma
HDAC(i): histone deacetylase (inhibitor)
SAHA: suberoylanilide hydroxamic acid
SCFA: short chain fatty acid
TSA: Trichostatin A
VPA: valproic acid

## Introduction

It is now almost 40 years since the first demonstration that treatment of cells in tissue culture with salts of butyric acid (a short chain fatty acid, SCFA) caused increased levels of histone acetylation 1. This was accompanied by slowed cell cycle progression 2 but no dramatic, overall change in RNA synthesis 3. It was shown that hyperacetylation resulted from inhibition of deacetylase activity 4. These early experiments demonstrated the rapid turnover of histone acetate groups, and provided an invaluable method for production of acetylated histones for structural and functional analyses. The observation was particularly exciting in view of the possible link between histone acetylation and transcription 5, and that low concentrations of butyrate could induce differentiation of an erythroleukemia cell line into non‐dividing, hemoglobin synthesizing cells 6.

These experiments all predated elucidation of the role of histones in DNA packaging, identification of enzymes responsible for turnover of histone acetates, formulation of the histone code hypothesis and realization of the extraordinary complexity of the processes by which histone post‐translational modifications regulate chromatin function (reviewed in 7, 8). But despite the vastly increased amount of information at our disposal, and increasingly sophisticated technologies by which to generate even more, fundamental questions posed nearly 40 years ago about the functional role of histone acetylation, remain unanswered 9. The problem is particularly acute in view of the increasing clinical use of histone deacetylase inhibitors (HDACi), both simple SCFA salts such as sodium valproate, and more complex reagents.

In this article, we ask how it is that most normal cells are able to tolerate the extreme hyperacetylation of histones and other proteins induced by HDACi and why just a few types of cancer are so sensitive. If histone acetylation really is an essential component of transcription control mechanisms, then its disruption should be consistently lethal. To answer this, we present recent evidence that cells have an intrinsic, chromatin‐based transcriptional response that mitigates the potentially deleterious effects of HDACi. We present an evolutionary rationale for the existence of such a response, and speculate on how HDACi might mediate chemical interactions between divergent organisms. Predicting how any particular cell, whether normal or cancerous, is likely to respond to HDACi, is an issue with obvious relevance for improving the clinical effectiveness of this family of inhibitors.

## Histone deacetylase inhibitors are effective in treating certain cancers

In 2006, the US Food and Drug Administration (FDA) approved the use of a histone deacetylase inhibitor, suberoylanilide hydroxamic acid (SAHA, marketed by Merck as Zolinza), for the treatment of cutaneous T‐cell lymphoma (CTCL). The drug is remarkably effective against this rare cancer, as is the structurally unrelated inhibitor depsipeptide, a bicyclic peptide marketed as Romidepsin. More recently, two further hydroxamic acid HDACis have also been granted FDA approval, Belinostat against peripheral T‐cell lymphoma and Panobinstat, against multiple myeloma. These successes show that HDACi, as a class of drugs, can be effective chemotherapeutic agents 10, 11.

Recently, there has been a proliferation of trials testing HDACi against a variety of cancers, often in combination with other drugs, such as aza‐cytidine (a DNA methyltransferase inhibitor) and Bortezomib (a proteasome inhibitor) 10, 12. Inhibitors for which completed trials have been reported, and the cancers against which they have been tested, are listed in Tables 1 and 2. Although hematological malignancies remain the major clinical targets for HDACi, several have now been trialed against more common solid tumors, including lung, breast and prostate cancer (Tables 1 and 2). Results have been mixed; the variability of response, both between and within cancer types is striking, and remains unexplained 13.

**Table 1:** Histone deacetylase inhibitors in clinical trials against cancer

Structural class	Name	HDACs inhibited*	Cancer type	Clinical trial status
Benzamide	4SC‐202	I	AHM	I
	Chidamide (Epidaza, HBI‐8000)	HDAC1,2,3,10	PTCL	Approved (Chn)
			NSCLC	II
	CI994	HDAC1,3	NSCLC	III
			MuMy, Pan	II
	Entinostat (SNDX‐275)	HDAC1,2,3,9	AML, Br, Col, MDS, MM, NSCLC	II
			ALL, Lym	I
	Mocetinostat (MGCD0103)	HDAC1,2,3,11	CLL, Lym, MDS	II
			NHL	I
Carboxamide	Abexinostat (PCI‐24781)	HDAC1,2,3,6,8,10	Sar, Lym	II
	Quisinostat (JNJ‐26481585)	I,II,IV	CTCL	II
			Lym, MuMy, NSCLC, Ov	I
Cyclic peptide	Depsipeptide (Romidepsin)	HDAC1,2,4,6	CTCL, PTCL	Approved (US)
			AML, Br, Col, Gli, MDS, MuMy, NHL, NSCLC, RCC, SCLC, Thy	II
			Lym, Pan	I
Hydroxyacrylamide	Resminostat (4SC‐201/RAS2410)	HDAC1,3,6	Col, HCC, HL	II
Hydroxamic acid	Belinostat (PXD101)	I,II	PTCL	Approved (US)
			AML, CTCL, Liv, NLH, MDS, Mes, MuMy, NHL, NSCLC, Ov, Sar, Thy	II
			AHM, Lym	I
	CUDC‐101	I,II (+EGFR, HER2)	Br, Gas, HNC, Liv, NSCLC	I
	Givinostat (ITF2357)	I,II	HL, MuMy, PCV	II
	Panobinostat (LBH589)	I, II	MuMy	Approved (US)
			CTCL, HL	III
			Br, MDS, Pr, TCL	II
			ALL, AML, CML, Col, MCL, NHL	I
	Pracinostat (SB939)	I,IIa, HDAC6, IV	AML, MDS, Pr, Sar	II
	SHP‐141	Not available	CTCL	I
	Tefinostat (CHR‐2845)	Not available	AHM	I
	Trichostatin A	I, II		
	Vorinostat (SAHA)	I, IIa, IIb, IV	CTCL	Approved (US)
			Mes, MuMy	III
			AML, ALL, Br, GBM, HL, MDS, MM, NHL, NSCLC, Pr, RCC, Sar	II
			APL, Col, DLBCL, NBM, Pan	I
Isothiocyanate	Sulforaphane (brocolli)	Not available	Br, Pr	II
Short chain fatty acid	Pivanex (AN‐9)	I, IIa	NSCLC	II
	Valproic acid	I, IIa	AML, HNC, MDS, Mes, Sar, SCLC	II
			CLL, Lung, MDS, NSCLC, Ov, SLL	I

Inhibitors are grouped by their basic chemical structure. The HDACs against which specific inhibitors act are shown, where known, along with the cancers against which they are being trialed and the furthest stage of trial reached.

*HDAC classes: Class I, HDAC 1, 2, 3, 8; Class IIa, HDAC 4, 5, 7, 9; Class IIb, HDAC 6, 10; Class III, SIRT1‐7 (these NAD‐dependent deacetylases are not inhibited by HDACi); Class IV, HDAC 11. Source: ClinicalTrials.gov (NIH)

**Table 2:** Abbreviations of cancer types used in Table 1 and the number of HDACi compounds which have completed trials or are approved for each cancer

Abbreviation	Cancer	*n*	Abbreviation	Cancer	*n*
AHM	Advanced hematological malignancies	3	MCL	Mantle cell lymphoma	1
ALL	Acute lymphoblastic leukemia	3	Mes	Mesothelioma	3
AML	Acute myeloid leukemia	7	MM	Malignant melanoma	2
APL	Acute promyelocytic lymphoma	1	MuMy	Multiple myeloma	7
Br	Breast	6	NBM	Neuroblastoma	1
CLL	Chronic lymphocytic leukemia	1	NHL	Non‐hodgkins lymphoma	5
CML	Chronic myeloid leukemia	1	NSCLC	Non‐small cell lung cancer	10
Col	Colorectal	5	Ov	Ovarian	3
CTCL	Cutaneous T‐cell lymphoma	6	Pan	Pancreatic	3
DLBCL	Diffuse large B‐cell lymphoma	1	PCV	Polycythemia vera	1
Gas	Gastric cancer	1	Pr	Prostate	4
GBM	Glioblastoma multiforma	1	PTCL	Peripheral T‐cell lymphoma	3
Gli	Glioma	1	RCC	Renal cell carcinoma	2
HCC	hepatocellular carcinoma	1	Sar	Sarcoma	5
HL	Hodgkins lymphoma	3	SCLC	Small cell lung carcinoma	1
HNC	Head and neck cancer	2	SLL	Small lymphocytic lymphoma	1
Liv	Liver	2	TCL	T‐cell lymphoma	1
Lung	Lung	1	Thy	Thyroid	2
Lym	Lymphoma	6			

Understanding how HDACi affect different cell types is complicated by the fact that protein deacetylation is carried out by a family of enzymes, within which individual members differ in their sensitivities to different HDAC inhibitors. Fortunately, a substantial body of research since purification and characterization of the first HDAC in 1996 14 has provided valuable insights into the HDAC family and the factors that regulate its activities.

## HDACs are a diverse enzyme family whose activities depend on associated proteins

There are 18 different HDACs in human cells, split into four classes (reviewed in 15). Eleven of these enzymes, classes I, IIa, IIb, and IV, have a very similar catalytic site. Class III enzymes, the sirtuins, are NAD dependent, and are insensitive to all classes of HDACi in clinical use 15, 16. It is important to remember that HDACs, despite their name, act on a variety of proteins in addition to histones, including transcription factors, enzymes and HDACs themselves 17.

Class I HDACs are nuclear enzymes that are likely to be involved in maintaining histone acetylation levels. The catalytic activities of class IIa enzymes are very low in vitro and seem to be unnecessary for at least some of their in vivo functions 18. They shuttle between the cytoplasm and nucleus and may play a structural role in complexes involved in regulating chromatin function 19. The class IIb enzymes act primarily on cytoplasmic components while the only class IV enzyme, HDAC11, is present in low amounts and is of uncertain function 20. It is important to note that levels of individual HDACs vary widely from one tissue to another and the HDACs that constitute the most promising targets for therapy may depend on the tissue in which the tumor originated.

Attempts have been made to establish the roles of the different HDACs in processes relevant to cancer treatment, such as growth control, differentiation, and apoptosis. Knocking out HDACs 1–4, 7, and 8 in mice is lethal during development (E9.5–11) or the early post‐natal period, while knockouts of HDACs 5, 6, and 9 are viable 20. Surprisingly, complete deletion of any one of the class I HDACs had no major effect on the growth or viability of tumor cell lines, despite their in vivo lethality 21. However, knockout of both HDAC 1 and 2 resulted in rapid cell death, largely as a result of aberrant mitosis. Significantly, HDAC 1/2 ‐/‐ fibroblasts transformed with H‐Ras, did not form tumors when injected into nude mice 21. These experiments show that HDACs 1 and 2 are key, redundant targets for suppression of tumor growth by HDACi.

HDACs1–3 are catalytically active only when physically associated with specific partner proteins. Four such HDAC complexes have been isolated and characterized, namely CoRest, NuRD, Sin3 (each containing HDACs 1 and 2) and NCoR/SMRT, which contains HDAC3 22. The complexes are responsible for the up‐ and/or down‐regulation of specific genes and are necessary for the correct differentiation of various cell types 23. Further complexity is introduced by structural studies showing that HDAC activity of the NCoR and NuRD complexes requires incorporation of a molecule of D‐myo‐inositol 1,4,5,6 tetrakisphosphate (ins(1,4,5,6)P4), a crucial mediator of intracellular signaling pathways 24. A recent paper has introduced a further level of HDAC regulation by demonstrating complementary roles for monomeric and polymeric nuclear actin in regulation of HDAC1 and 2 25.

Thus HDACs, collectively, have multiple substrates and are regulated at multiple levels, including association with other proteins, chemical modification, and interaction with components of intracellular signaling pathways.

## HDACi generally inhibit several members of the HDAC family

In view of the high degree of conservation of the HDAC catalytic site between all but the class III (NAD dependent) HDACs, it is not surprising that most inhibitors target more than one family member. The specificities of the inhibitors currently in trials are shown in Table 1. This broad specificity makes it difficult to interpret the results of inhibitor treatment in terms of precise biochemical mechanisms. Attempts have been made, with some success, to synthesize derivatives specific for individual HDACs and one such (a benzamide, 4SC‐202) 26, 27 is in a phase I clinical trial (Table 1). However, the functional redundancy of HDACs, particularly class I enzymes 20, 21, may reduce the clinical effectiveness of enzyme‐specific inhibitors. It is also worth noting that the specificities of individual HDACs have been determined by using pure, recombinant enzymes against artificial substrates. The catalytic activity of recombinant HDACs is extremely low and unlikely to reflect the in vivo situation where the enzyme itself can be part of different multi‐protein complexes and exposed to the various factors (such as inositol phosphates) that modify its activity. An indirect, mass‐spectrometry‐based assay has been devised to examine the in vivo sensitivities of the different HDAC complexes to common inhibitors 28. Though technically challenging, such approaches may provide a deeper understanding of HDAC inhibitor sensitivities.

## Why most cells are tolerant of HDACi‐induced hyperacetylation

A recent paper from the authors’ laboratory 29, addresses the question of why cells are usually so tolerant of HDACi‐induced protein hyperacetylation. How do they deal with the disruption of fundamental cell functions that would be expected to ensue? To address this, attempts were made to identify the earliest transcriptional and epigenomic responses to HDACi. Human lymphoblastoid cells were exposed to two chemically different HDACi (SAHA and sodium valproate) for 30, 60, and 120 minutes At this early stage, and for both inhibitors, cells were found to undergo a progressive and coordinated change in expression of a small and distinctive set of genes. Growth‐promoting cytokines were down‐regulated, presaging a later slowing of cell growth, while there was a rapid up‐regulation of genes encoding DNA binding proteins and transcription factors. But the most striking response was the consistent and severe down‐regulation of genes encoding components of the six enzyme complexes responsible for protein acetylation, the lysine acetyltransferases, KATs 17. The rapid depletion of KAT complex components inevitably diminishes the aberrant acetylation of histones and other proteins when the deacetylating enzymes are blocked. KAT and HDAC complexes have both been shown to target the acetylated promoters of active genes 30, so KAT depletion would be expected to counter the effects of HDACi at those loci where histone acetylation is associated with gene expression. We propose that this transcription‐based response allows the cell to reduce, and eventually reverse, protein hyperacetylation at critical regulatory regions, thereby reducing the epigenetic disruption caused by HDACi.

It is notable that, even at the earliest time points, there was no consistent association between increased histone acetylation at transcription start sites and increased gene expression. This indicates that histone hyperacetylation, at least at the three lysines studied so far, does not drive increased gene expression in this system, in agreement with previous studies 31, 32, 33, 34. However, it is striking that the genes that change expression in response to HDACi, whether up‐ or down‐regulated, are packaged in highly acetylated chromatin 29. It seems that histone acetylation helps provide a chromatin context within which genes are able to change their transcriptionl state in response to HDACi, and perhaps other regulatory signals.

Surprisingly, the most substantial change in histone modification we observed was an increase in the levels of the Polycomb‐associated silencing mark H3K27me3, specifically at transcription start sites and mostly at genes whose expression did not change 29.The change in H3K27me3 levels at TSS was shown to be required for the short term, HDACi‐induced changes in transcription at some loci. A chemical inhibitor of the enzyme responsible for H3K27 methylation, EZH2, prevented the up‐ or down‐regulation of selected groups of genes 29. How the changes in H3K27me3 are triggered, and how they influence the response to HDACi, are questions that require further investigation.

## Does a reversibly acetylated non‐histone protein control the response to HDACi?

**Figure 1:**
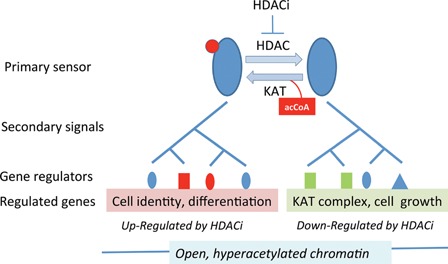
A hypothetical primary sensor and signaling cascade by which cells respond to environmental HDACi. The proposed primary sensor protein (blue ovals) is subject to acetylation (red disc) at one or more lysines. The balance between acetylated and non‐acetylated isoforms is determined by the activities of a lysine acetyltransferase (KAT) and deacetylase (HDAC). Each isoform is responsible for regulating the activities of a set of genes with distinctive functional associations (ontologies), as indicated (pink or green shading). It does this by initiating a series of secondary signals (a signaling cascade) that ultimately results in transcriptional activation at all target genes through induced (or activated) chromatin binding proteins (gene regulators, blue, green, or red shapes). HDAC inhibitors tip the balance of the primary sensor toward the acetylated isoform, resulting in increased expression of the genes under its control, with reduced expression of genes regulated by the non‐acetylated (now diminished) isoform. Genes up‐ or down‐regulated by HDACi have been shown to be packaged in hyperacetylated chromatin, presumably to maintain an open chromatin structure accessible to regulatory factors. The model is based on experimental data presented in Halsall et al. 29.

The lack of association between changes in histone acetylation and transcription over the early stages of HDACi treatment, suggests that histones themselves are not the primary driver of early transcriptional change. What is? In Fig. 1, we present a model in which the acetylated and non‐acetylated isoforms of a master regulator protein enhance the expression of different sets of genes. The relative expression levels of these genes are dependent on the balance between acetylated and non‐acetylated isoforms. HDACi cause the balance to shift dramatically toward the acetylated form, resulting in up‐regulation of one set of genes and down‐regulation of the other (Fig. 1). The response is self‐limiting in that the universal down‐regulation of KAT complex components by HDACi will diminish acetylation of the primary sensor, thereby increasing the non‐acetylated isoform and restoring transcription of KAT complex components (Fig. 1). As things stand, the model is entirely hypothetical. Experiments are needed to identify the primary sensor protein, the KAT and HDAC involved in its acetylation, the steps in the signal cascades that link the sensor to specific sets of genes and the chromatin‐binding proteins that finally mediate the observed changes in gene expression. Depending on the number of steps in the signal cascades, it is likely that multiple gene regulatory proteins are involved for both down‐ and up‐regulated genes (Fig. 1). Some proteins may be used for both sets of genes. It is important that both signal cascades are responsible for supporting transcription of their respective sets of target genes. Thus, the down‐regulation of KAT genes and others in response to HDACi, is due to diminution of this positive transcriptional control, rather than active down‐regulation.

The components of the signal cascades triggered by the primary sensor, and the chemical changes involved, remain to be determined, but could involve phosphorylation, mediated by kinases and phosphatases as in many other signaling pathways, or other reversible modifications, including protein acetylation. In the latter case, the involvement of Class III deacetylases would protect the cascade from disruption by HDACi, to which these enzymes are resistant 16. It has become clear that reversible lysine acetylation is a key regulator of enzyme function and metabolism in both eukaryotic cells and bacteria 35, 36, 37.

## HDACi are natural products that can kill or manipulate competing organisms

It is, at first sight, surprising that human cells have the ability to mount such a carefully calibrated response to various inhibitors of a specific enzyme family. However, the fact that so many HDACi are natural products, suggests an evolutionary rationale through which these findings are readily explained.

HDACi of various chemical shapes and sizes are secreted by a wide range of organisms, often to kill, or at least suppress the growth of, competing life forms, (reviewed by Salvador and Luesch 38). Some examples are presented in Table 3. Short chain fatty acids, all broad spectrum HDAC inhibitors, are prolific by‐products of bacterial metabolism 39, 40. Other, chemically more complex, HDACi seem to be specifically synthesized. The first hydroxamic acid‐based HDAC inhibitor to be identified, Trichostatin A (TSA), is secreted by selected species of bacteria and is an antifungal antibiotic 41, 42. Three types of bacterial HDACi based on a cyclic depsipeptide chemical backbone have been identified, FK228/Romidepsin 43, spiruchostatin 44 and largazole 45, 46. The organisms they act against remain to be identified, though fungi are likely targets (Table 3).

**Table 3:** Natural products that act as histone deacetylase inhibitors

Inhibitor	Chemical type	Source organism	Target organism	References
Butyrate et al.	Short chain fatty acid	Most bacteria	Eukaryotic cells	4
Trichostatins	Hydroxamic acid derivatives	Bact; *Streptomyces hygroscopicus*	Fungi (*Trichophyton*, *Aspergillus*)	41, 42
FK228 (Romidepsin)	Cyclic depsipeptide	Bact; *Chromobacterium violaceum*	Fungi?	43
Spiruchostatin	Cyclic depsipeptide	Bact; *Pseudomonas chloroaphilis*	Fungi?	44
Largazole	Cyclic depsipeptide	Marine cyanobacterium (*Symploca* sp)	unknown	45, 46
Depudecin	Linear polyketide	Fungus (*Alternaria brassicicola*)	unknown	48
HC toxin	Cyclic tetrapeptide	Plant fungus (*Cochliobolus carbonum*)	Green plants	47, 49
Trapoxins	Cyclic tetrapeptide	Fungus (*Helicoma ambiens*)	unknown	50
Apicidin	Cyclic tetrapeptide	Fungus (*Fusarium pallidoroseum*)	Apicomplexan parasites	51, 52, 53
Azumamides	Cyclic tetrapeptide	Marine sponge (*Mycale izuensis*)	unknown	54
Psammaplin A	Linear bromotyrosine	Marine sponge (*Psammaplysilla sp*)	unknown	55, 56
Sulforaphane	Isothiocyanate	Cruciferous plants (eg broccoli)	Pathogenic fungi?	58, 59

The table shows the basic chemical structures of naturally occurring HDACi, organisms that make them and, where known, the organisms against which they act in vivo. Detailed chemical structures for the inhibitors listed can be found in the review by Salvador and Luesch 38.

Most fungal species seem to be resistant to HDACi, though mechanisms of resistance have only rarely been reported 47, and some even make their own. Examples include the linear polyketide depudecin 48 and the cyclic tetrapeptides HC‐toxin 47, 49, and Trapoxin 50. Another fungal HDACi, apicidin (a cyclic tetrapeptide made by *Fusarium* spp.) specifically kills some epicomplexan parasites (e.g. *Plasmodium berghei*) 51, 52. In a survey of 52 *Fusarium* isolates, only one produced apicidin 53. Some more complex, multicellular eukaryotes also secrete HDACi. Marine sponges synthesize HDACi based on either a cyclic tetrapeptide backbone (azumamides) 54 or bromotyrosine (Psammaplin A) 55, 56, 57, and may serve to deter micro‐organisms sharing the same aquatic environment. The isothiocyanate‐based HDACi sulforaphane, is produced by cruciferous plants such as broccoli 58 and first became of interest because of its ability to suppress the growth of transformed cells in tissue culture 58, 59, 60. It remains to be shown how this inhibitor, and HDACi in general, might be involved in the cancer‐preventing effects of cruciferous vegetables and other dietary components 60, 61, 62, 63.

Competition for space and resources in the microbial world is often intense, and it is no surprise that an almost infinite variety of coping strategies have evolved. The energy and resources that some organisms devote to synthesizing sometimes chemically complex deacetylase inhibitors is presumably justified by the selective advantage they confer. The processes by which bacterial HDACi kill or deter competing organisms remain to be established, but it is likely to be important that fungi and protists (eukaryotes) have chromatin‐based epigenetic control systems that bacteria (prokaryotes) lack.

## Eukaryotic chromatin‐based control systems provide a likely target for manipulation by prokaryotes

All life forms on earth consist of one or another of just two cell types, prokaryotic (bacteria and archaea) and eukaryotic (everything else, including all complex multicellular life forms). Though derived from a common ancestor 64, the two cell types are qualitatively different. Eukaryotes are classically distinguished by possession of a nuclear envelope, microtubules, and mitochondria (or equivalent) 65. In addition, although prokaryotes have histone‐like proteins that bind DNA 66, only eukaryotes package their DNA as chromatin, which invariably is, or once was 67, based on the canonical eight‐histone nucleosome core particle 7, 68. Thus, chromatin, nucleosomes and epigenetic control systems based on chromatin modifications, are uniquely eukaryotic. In this respect, they are likely targets for competing prokaryotes. We suggest that the HDACi secreted by some bacteria provide one example of this targeting.

Eukaryotes would be expected to have evolved responses to environmental HDACi, and we suggest that the transcription‐based resistance mechanism present in human cells is an example of this 29. As a competitive strategy, the secretion of HDACi will succeed only while the competing species are susceptible. Once they become resistant, new strategies are required. This may explain why most bacteria do not secrete, or are not known to secrete, HDACi, and why most eukaryotes seem resistant to their effects.

**Figure 2:**
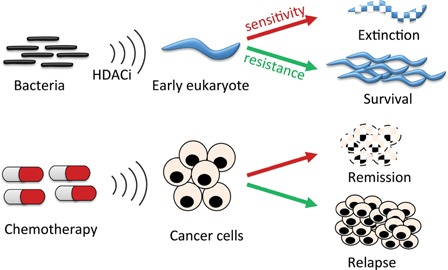
How a resistance response to histone deacetylase inhibitors (HDACi) might influence both evolution of eukaryotes and response of tumor cells to chemotherapy. We propose that eukaryotes have evolved a response that allows them to deal with the hyperacetylation caused by environmental (often bacterial) HDACi (upper part). Cancer cells in which this response remains intact can resist chemotherapeutic HDACi, whereas those in which the response has been compromised, either by mutation or through additional drug treatment, are killed, leading to a period of remission (lower part).

The resistance response to HDACi may be evolutionarily very ancient. It is estimated that the first prokaryotic life forms emerged about 3.5 billion years ago, 1 billion years or so after the planet was formed 69. The emergence of the first eukaryotes is hard to establish, but is unlikely to be more than 2 billion years ago, perhaps much less 70. The first eukaryotes emerged into ecosystems dominated by prokaryotes, who would have used all means at their disposal to see off the new competitors; targeting their evolving, but uniquely eukaryotic, chromatin, and epigenetic signaling networks would be a promising approach (Fig. 2).

Evolution generally proceeds by small steps, by selection of small mutational changes to existing systems. The development, by early eukaryotes, of defensive strategies to cope with prokaryotic HDACi, must have proceeded in the same way. In this respect, the simple model presented in Fig. 1 suggests a possible evolutionary pathway for the establishment, and progressive improvement, of a resistance response. All cells have systems by which they maintain metabolic homoeostasis in the face of environmental change and their own growth and reproductive cycles. The signal cascades shown in Fig. 1 could be viewed as components of a homeostatic system for maintenance of levels of a key metabolite, acetylCoA. The intracellular concentration of acetylCoA has been shown to regulate levels of protein acetylation 36, 71, and the primary sensor postulated in Fig. 1 could have evolved from a sensor used to monitor the concentration of acetylCoA and regulate expression of genes encoding acCoA‐producing or or acCoA‐metabolising enzymes.

It would be wrong to assign the prokaryote‐eukaryote interactions proposed here to the evolutionary past. We live in a world in which prokaryotes are ubiquitous, and even, by some measures, the predominant life form 72. The human body is host to a variety of bacterial species, some of which are closely involved in key physiological functions, and are disrupted in disease 73. For example, colonic bacteria give rise to millimolar concentrations of salts of various short chain fatty acids in the mammalian large intestine 74, 75, 76, 77. Cells of the colonic epithelium, including differentiating stem cells involved in replacement of the surface layer, must therefore accommodate inhibitory concentrations of bacterial HDACi. Given the subtlety with which prokaryotes can influence patterns of gene expression in neighbors through chemical signals 78, this is unlikely to be the only situation in which endogenous micro‐organisms influence our epigenetic systems 73. Nor is it likely that HDACs are the only targets.

## Conclusions

This article began with a description of the potential value of HDACi as chemotherapeutic drugs, progressed through the possible influences of HDACi in the co‐evolution of prokaryotic and eukaryotic micro‐organisms, and has finished by highlighting the ongoing chemical interactions between complex eukaryotes (including ourselves) and the microbial world. This summary returns to where we started, by noting that the existence of a resistance response to HDACi, and its evolutionary provenance, has clinical implications. Certain cancers may be sensitive to HDACi because their resistance mechanism has been disrupted (Fig. 2), perhaps by mutation of one or more of its essential genes, or components of the (as yet hypothetical) primary sensor or signal cascade (Fig. 1). Identification of such mutations will help target individual cancers susceptible to HDACi. Similarly, combining HDACi with drugs that undermine the resistance mechanism specifically in cancer cells, may open the way to more successful treatment of currently refractory cancers through combination therapy. It will also be interesting to search for other pathways by which prokaryotic metabolites might dysregulate epigenetic control systems peculiar to eukaryotes, and to ask by what means eukaryotes defend themselves against such manipulation. Understanding these interactions may be crucial in maximizing the clinical benefit of the many chemotherapeutic drugs based on natural products.

The authors have declared no conflicts of interest.
